# Psychological job strain, social support at work and daytime secretion of dehydroepiandrosterone (DHEA) in healthy female employees: cross-sectional analyses

**DOI:** 10.1038/srep15844

**Published:** 2015-11-10

**Authors:** Atsuhiko Ota, Hiroshi Yatsuya, Junji Mase, Yuichiro Ono

**Affiliations:** 1 Department of Public Health, Fujita Health University School of Medicine, Toyoake, Japan; 2 Division of Dentistry, Aichi Cancer Centre Hospital, Nagoya, Japan

## Abstract

Evidence is limited concerning the influences of high psychological job strain and low social support at work on daytime secretion of dehydroepiandrosterone (DHEA), which demonstrates anti-cortisol effects. We carried out a cross-sectional study to examine the associations of job strain and social support with daytime secretion amounts of DHEA and cortisol and daytime variation of the cortisol-to-DHEA ratio (C/D ratio) in healthy female workers. Study subjects comprised 115 healthy female nursery school teachers. Area under the curve with respect to ground (AUC_G_) of salivary DHEA, cortisol and C/D ratio was calculated for estimation of daytime secretion and variation. Social support scores were negatively associated with daytime DHEA secretion (standardized partial regression coefficient = −0.343, P < 0.001 by multiple linear regression analysis). This association remained significant when daytime cortisol secretion was additionally adjusted. Social support was not associated with daytime variation of the C/D ratio. Significant association between social support and daytime cortisol secretion was not confirmed. Job strain was not associated with DHEA, cortisol or the C/D ratio. In summary, we found that daytime DHEA secretion was increased in healthy workers with low social support, perhaps independent of daytime cortisol secretion.

The Demand–Control–Support model (DCS model)^1^ is a theory-based conceptual model for assessment of adverse psychosocial job stressors. In the model, high psychological job strain is defined as a condition in which quantitatively high and conflicting demands are combined with little decision authority and skill utilization. Social support at work is defined as positive, helpful social interaction with supervisors and co-workers in the workplace. Epidemiological research has shown that high job strain and low social support could be a risk factor or an effect modifier in the development of coronary heart disease^2^,^3^, blood pressure elevation^4^,^5^,^6^, mental disorders^7^,^8^,^9^ and musculoskeletal disorders in the neck and shoulders^10^,^11^.

The underlying physiological mechanism of the relationship of high job strain and low social support with adverse health disorders remains unclear. Some researchers’ idea was that, like acute stress responses^12^, chronic exposure to high job strain and low social support might stimulate the hypothalamic–pituitary–adrenocortical (HPA) axis and increase cortisol secretion. It has been reported that increased cortisol secretion and dysfunction of the HPA axis could play a significant role in the development of coronary heart disease^13^, depression^14^,^15^,^16^,^17^ and musculoskeletal pain^18^. Associations of the HPA activity with visceral adiposity^19^,^20^ and immune reaction^14^,^21^,^22^ may support these findings. In this context, a large number of studies have been conducted to examine whether high job strain and low social support increase daytime cortisol secretion^23^,^24^,^25^,^26^,^27^,^28^,^29^,^30^,^31^,^32^,^33^,^34^, but the results are conflicting.

Only a few studies have evaluated dehydroepiandrosterone (DHEA)^35^,^36^,^37^ to determine the physiological mechanism underlying chronic stress responses to high job strain and low social support. DHEA is a steroid mainly synthesized in the adrenal cortex. DHEA is sulphated (DHEAS) before entering the circulatory system. DHEAS is converted back to DHEA by steroid sulphatase. DHEA demonstrates anti-glucocorticoid, i.e., anti-cortisol, effects. Cortisol-to-DHEA ratio (C/D ratio) or cortisol-to-DHEAS ratio would be a better measurement than that of cortisol alone in a functional evaluation of hypercortisolemia^35^,^36^. Existing evidence is limited concerning the relationship of job strain and social support with DHEA, C/D ratio and cortisol-to-DHEAS ratio. A study of female hospital personnel did not find a significant association between job strain and fasting morning plasma DHEA^38^. Hansen *et al.*^39^ showed that social support was not significantly associated with morning plasma DHEAS in female sewing machine operators. While job strain was associated with DHEAS and cortisol-to-DHEAS ratio in management personnel, Gadinger *et al.*^40^ reported that the dimensions of the DCS model did not predict overnight urinary cortisol, fasting morning plasma DHEAS or cortisol-to-DHEAS ratio in non-management personnel. As previous studies have only measured morning DHEA or DHEAS levels, it is unclear whether high job strain and low social support affect daytime secretion of DHEA and daytime variation of the C/D ratio.

Considering the pharmacological effects of cortisol and DHEA, we speculate that a compensatory increase in daytime DHEA secretion may occur in healthy workers to suppress the effects of increased daytime cortisol secretion due to high job strain and low social support: as a result, the C/D ratio remains constant. In the present cross-sectional study, we examined the associations of job strain and social support with daytime secretion amounts of DHEA and cortisol and daytime variation of the C/D ratio in healthy Japanese female nursery school teachers.

## Results

We cross-sectionally analysed the baseline dataset of a prospective cohort study which was conducted to clarify the relationship between work-related psychosocial factors and musculoskeletal disorders in nursery school teachers^41^. Table 1 presents general characteristics (age, employment status, current smoking, menstruation irregularity, ovulatory phase and health disorders), job strain and social support scores and logarithmic (log)-transformed area under the curve with respect to ground (AUC_G_)^42^ of salivary DHEA, cortisol and C/D ratio of the subjects. The subjects comprised 115 healthy female nursery school teachers, aged between 20 and 49. Subjects took part in the current study on a voluntary basis and were not pregnant or taking medicine that could affect cortisol and DHEA secretion. None of the subjects worked at a managerial position (Supplementary Figure S1). Those with higher job strain scores were assumed to be burdened by higher psychological job demand along with lower skill discretion and/or decision latitude. Those with lower social support scores were supposed to be working with less supportive supervisors and co-workers. AUC_G_ of salivary DHEA, cortisol and C/D ratio was calculated to estimate daytime secretion amounts of DHEA and cortisol and daytime variation of the C/D ratio. Salivary DHEA and cortisol levels and C/D ratio at 9:00, 12:00 and 15:00 were used for the calculation (Table 2). AUC_G_ was log-transformed for normality.

We first computed Pearson correlation coefficients to examine the associations of job strain and social support scores with log-transformed AUC_G_ of salivary DHEA, cortisol and C/D ratio (Table 3). Job strain did not correlate with DHEA level, cortisol level or the C/D ratio. Social support scores negatively correlated with DHEA. There was a positive correlation between DHEA and cortisol. We also examined the bivariate associations between general characteristic variables and log-transformed AUC_G_ of salivary DHEA, cortisol and C/D molar ratio (Table 3 & Supplementary Table S2). Age correlated negatively with DHEA level and positively with the C/D ratio. There was a significant correlation between employment status and DHEA. The regular staff showed larger log-transformed AUC_G_ of salivary DHEA than the contract workers (mean [standard deviation]: 0.32 [0.56] vs. 0.02 [0.70], P = 0.021 by *t*-test). The remaining general characteristic variables were not associated with salivary DHEA, cortisol or C/D ratio (Supplementary Table S2).

Next, using multiple linear regression analyses, we calculated standardized partial regression coefficients (SPRCs) of job strain and social support scores for log-transformed AUC_G_ of salivary DHEA, cortisol and C/D ratio (Table 4). We made two kinds of models. In Model 1, independent variables were job strain and social support scores, age and employment status. Goodness of fit for all regression models was significant. Adjusted R^2^ of the regression model was very low for cortisol. Job strain was not associated with DHEA, cortisol or the C/D ratio. Social support scores were negatively associated with DHEA and cortisol. The association between social support and DHEA remained significant even when log-transformed AUC_G_ of salivary cortisol was additionally included in the independent variables (Supplementary table S3). There was no significant association between social support score and the C/D ratio. In Model 2, independent variables included the remaining general characteristic variables additionally. The results for DHEA and the C/D ratio changed little. In contrast, formula regression of cortisol and the association between social support and cortisol lost statistical significance. Multicollinearity was absent in all the regression formulae.

## Discussion

Present findings showed that low social support was associated with an increase in daytime secretion amount of DHEA, but was not associated with daytime variation of the C/D ratio. The association between low social support and an increase in daytime secretion amount of DHEA remained significant even after daytime secretion amount of cortisol was adjusted. Simultaneously, low social support exhibited an inconsistent significant association with an increase in daytime cortisol secretion amount. Job strain was not associated with DHEA, cortisol or the C/D ratio. When interpreting the present results, we have to keep in mind that we examined healthy workers. That is, we observed what was happening during the pre-clinical phase. The associations of job strain and social support with daytime DHEA secretion may be different in the clinical phase. In addition, only with the present findings, we cannot prove a compensatory increase in daytime DHEA secretion in response to chronic exposure to low social support because of the study design, i.e., cross-sectional examination. Strictly saying, the precise chronological order of exposure to low social support and an increase in daytime DHEA secretion cannot be determined in the present study.

Present results displayed an association between low social support and an increase in daytime DHEA secretion. Experimental studies showed an increase in DHEA level in response to acute psychosocial stress exposure^43^,^44^. A similar increase in daytime DHEA secretion may occur in response to chronic exposure to low social support. The present findings were not concordant with those of previous studies. Hansen *et al.*^39^ did not find a significant association between social support and morning plasma DHEAS in female sewing machine operators. The difference in time of sample collection between our study and theirs may have contributed to the different results obtained. To some extent, the variations in daytime secretion between DHEA and DHEAS may be explained by variations in DHEA and DHEAS secretion. DHEA secretion exhibits a diurnal variation, whereas DHEAS shows no or a small variation^45^. Moreover, the subjects’ work environment should be considered. For nursery school teachers, good communication with and assistance from supervisors and co-workers are indispensable. Thus, they may feel more burdened with chronic exposure to low social support at work than workers in more independent job environments. Gadinger *et al.*^40^ also reported that social support was not significantly associated with fasting morning plasma DHEAS in non-management personnel. They mentioned the heterogeneity of the participants’ work tasks and situations as a study limitation that reduced statistical power. Our study maintained homogeneity concerning the occupation of the subjects: all subjects were nursery school teachers.

Association between social support and the C/D ratio was absent in the current study. This finding may be supported by a recent Japanese study by Izawa *et al.*^46^, which examined the effects of a 2-week teaching practice at a kindergarten. The practice was regarded as a chronic psychosocial stressor on the HPA axis and DHEA secretion in healthy female students. Salivary cortisol, DHEA levels and C/D ratios were examined two weeks before, during and a few days after the practice. Unlike cortisol and DHEA levels showing variations, the C/D ratios remained constant throughout the observation period. Therefore, we speculate that some physiological mechanism exists in healthy young women to balance the C/D ratio against chronic exposure to psychosocial stressors.

We found a positive correlation between daytime secretion amounts of cortisol and DHEA in our study. At the same time, the association between low social support and cortisol was not statistically consistent, as will be discussed in the next paragraph. The association between social support and daytime DHEA secretion remained significant even when log-transformed AUC_G_ of salivary cortisol was additionally adjusted. We speculate that the increase in daytime DHEA secretion would not be accompanied only by increased daytime cortisol secretion. It could possible that chronic exposure to low social support was associated with daytime DHEA secretion, independent of increased daytime cortisol secretion, during the pre-clinical phase. Besides anti-glucocorticoid effects, DHEA demonstrates neuroprotection, catecholamine synthesis and secretion, anti-oxidant effects and anti-inflammatory effects^36^. These biologically beneficial effects may help female workers with low social support stay healthy during the pre-clinical phase. Further research is necessary to examine the underlying physiological mechanism of the relationship between low social support and DHEA secretion.

As previously mentioned, we failed to confirm the association between low social support and an increase in daytime cortisol secretion. Multiple linear regression formulae inconsistently presented statistical significance with regard to the association and explained the cortisol secretion only very slightly. One possible reason for these unexpected findings is that we only examined healthy workers. The association between social support and cortisol secretion may be more prominent in individuals in the clinical phase. Another reason is that daytime cortisol secretion might be more regulated by other psychosocial stressors such as impairment of work–life balance and family-related distress.

Job strain was not associated with DHEA, cortisol or the C/D ratio. This is concordant with the existing evidence^38^,^40^. We reported that effort–reward imbalance and overcommitment to work, as derived from the Effort–Reward Imbalance (ERI) model^47^, were not associated with daytime cortisol or DHEA secretion in the same subjects^41^. As previously mentioned, existing research presents contradictory findings on the associations between the DCS model dimensions and daytime cortisol secretion^23^,^24^,^25^,^26^,^27^,^28^,^29^,^30^,^31^,^32^,^33^,^34^. We are convinced that not all kinds of psychosocial work stressors affect the HPA axis or DHEA secretion in the same way. In fact, some studies^48^,^49^ showed a decrease in DHEAS secretion at morning and in response to acute stress exposure among those who were highly stressed over at work. There are some technical differences between these and our studies. In these studies, the researchers did not quantify daytime DHEA secretion amount or adopt the DCS or ERI model to assess psychosocial work stressors. However, these results could be inconsistent with our present results and call for further research to identify psychosocial work stressors which affect the HPA axis and DHEA and DHEAS secretion. Perhaps, how long employees are exposed to psychosocial work stressors may also be a possible determinant of the HPA activity and DHEA and DHEAS secretion. It is often difficult to measure the duration exactly. Moreover, different underlying mechanisms such as the autonomic nervous system^12^,^50^ and immune system^51^ must be as well accessed to determine the aetiology of health disorders related to psychosocial work stress.

Some study limitations may suggest caution when interpreting our results. First, since subjects were enrolled on a voluntary basis, it is possible that some were already health conscious and took preventive measures against psychosocial work stressors, such as exercise. This could mitigate the effects of high job strain and low social support on the HPA axis and DHEA secretion^52^. Moreover, the subjects may not be representative of female employees in Japan, limiting the generalizability of the present findings. Second, our saliva collection method also poses a potential limitation. We only collected saliva samples three times, at 9:00, 12:00 and 15:00, in a single working day. Daytime salivary cortisol and DHEA secretion amounts could be more accurately estimated by collecting saliva samples more often over multiple days. Finally, we did not collect information regarding alcohol consumption, diet, sleep (awakening time, sleep duration, sleep quality, etc.), education level or marital status, all of which could possibly affect the HPA activity and DHEA secretion^41^,^50^.

In conclusion, low social support was associated with an increase in daytime DHEA secretion amount and was not significantly associated with daytime variation of the C/D ratio in healthy female nursery school teachers. The association between low social support and an increase in daytime DHEA secretion was significant, independent of increased daytime cortisol secretion. Further prospective research is necessary to determine whether the increase in daytime DHEA secretion is compensatory in response to chronic exposure to low social support and for suppressing the effects of increased cortisol secretion.

## Methods

The present study was carried out in accordance with the Ethical Guidelines for Epidemiological Research established by the Ministry of Education, Culture, Sports, Science and Technology and the Ministry of Health, Labour and Welfare, Japan. The study was approved by the Ethics Review Committees of Fujita Health University, Japan (No. 11-098). The first author (A.O.) visited each nursery school to explain the study purpose and methods to eligible participants before recruiting the subjects. All subjects gave their written consent for participation in the study.

Study subjects comprised 115 female nursery school teachers. They were recruited on a voluntary basis from 29 nursery schools in Aichi prefecture, Japan (Supplementary Figure S1). Of the 503 employees, 158 participated in the present study. A total of 43 participants were excluded because they met one or more of the following exclusion criteria: age of 50 or older (n = 8), not being a nursery school teacher (such as a manager, cook and nurse) (n = 30), being pregnant (n = 1) and taking medication that could affect cortisol and/or DHEA secretion (n = 8). The remaining 115 were examined in the present study. The age range was set because of changes in DHEA and cortisol secretion related to aging and menopause^52^,^53^. Those who self-reported pregnant status were not eligible because pregnancy causes changes in the cortisol secretion mechanism^54^. Those who self-reported taking medication known to affect cortisol and/or DHEA secretion, such as oestrogen^55^, oral contraceptives^52^, steroids^52^,^56^ and antidepressants^16^,^52^, were excluded. Subjects completed a self-report questionnaire about general characteristics, job strain and social support and provided saliva for quantification of salivary cortisol and DHEA in a single working day from December to February of the years 2010–2012.

General characteristics included age, employment status (regular staff or contract worker), current smoking status, menstrual irregularity, ovulatory phase and health disorders (musculoskeletal symptoms, dental and gum diseases and other health problems). Subjects were regarded as having menstrual irregularity if they did not report regular menstruation. The authors estimated whether the subjects without menstrual irregularity were in the ovulatory phase. The estimation was based on their self-report of the length of the menstrual cycle and the day on which their last menstruation began. The half-length of the menstrual cycle was added to the beginning day of their last menstruation to estimate the ovulation day. Subjects were regarded as being in the ovulatory phase if the saliva collection day was within 3 days of the estimated ovulation day. Musculoskeletal symptoms included any subjective symptoms in the arms, hands, fingers or lower back that were annoying the subjects in their work. Subjects were asked whether they were under treatment for dental and gum diseases and other health problems.

Job strain and social support were scored with the Japanese version of the Job Content Questionnaire^57^,^58^. Originally, psychological job demand, control and social support scores are calculated with five, nine and eight items, respectively. Every item uses a four-point Likert scale. A job strain score is calculated by placing a demand score in the numerator and a control score in the denominator. The reliability and validity of the questionnaire has been reported elsewhere^57^,^58^. Cronbach alpha coefficient for control was low, 0.385, in the present study. It increased to 0.571 when a relevant item on repetitive work was excluded. Therefore, we excluded that item from calculation of control scores. Cronbach alpha coefficients for demand and social support were 0.613 and 0.906, respectively.

Subjects discharged 1 ml of saliva directly into a syringe at 9:00, 12:00 and 15:00 after rinsing their mouths with drinking water. We instructed them not to eat for 30 minutes before each saliva collection. Saliva samples were stored in a portable freezer at −25 °C immediately after collection. Cotton collection devices, such as Salivette, were not used. They are not recommended for an accurate quantification of salivary DHEA levels^59^,^60^.

Salivary cortisol and DHEA levels were quantified with the liquid chromatography-tandem mass spectrometry method performed by Aska Pharmaceutical Medical Co. Ltd. (Kawasaki, Japan). The technical details are described elsewhere^41^. A C/D ratio was calculated based on salivary DHEA and cortisol levels in units of nmol/l. AUC_G_ 42 of salivary DHEA, cortisol and C/D ratio was computed using the levels as collected at 9:00, 12:00 and 15:00. We expected that AUC_G_ of salivary DHEA and cortisol was an estimation of daytime secretion amount, while that of salivary C/D ratio was an indicator of daytime variation. Plasma and salivary concentrations are highly correlated for DHEA and cortisol^59^,^60^.

Statistical analyses were as follows: Pearson correlation test and *t*-test were used for bivariate analyses on the associations of job strain and social support scores and the general characteristic variables with log-transformed AUC_G_ of salivary DHEA, cortisol and C/D ratio. Log-transformation was employed for normality. Multiple linear regression analysis was adopted to examine the associations of job strain and social support scores with log-transformed AUC_G_ of salivary DHEA, cortisol and C/D ratio. Multiple linear regression formulae were constructed for each log-transformed AUC_G_ of cortisol, DHEA and C/D ratio as the dependent variable. Independent variables were job strain and social support scores and general characteristic variables. In Model 1, only job strain and social support scores, age and employment status (regular staff) were included because age and employment status were significantly associated with log-transformed AUC_G_ of DHEA and C/D ratio. In Model 2, the remaining general characteristic variables were additionally included. To analyse the association between social support and daytime DHEA secretion in more detail, log-transformed AUC_G_ of salivary cortisol was additionally included in the independent variables for both Models 1 and 2. To avoid multicollinearity, we considered the elimination of an independent that indicated the variance inflation factor being 10 or greater. Ultimately, we did not eliminate any independent variable. The level of significance was 0.05 (two-tailed) for all the tests. Statistical calculations were performed using IBM SPSS Statistics 22 Japanese version for Windows (IBM Japan, Tokyo, Japan).

## Additional Information

**How to cite this article**: Ota, A. *et al.* Psychological job strain, social support at work and daytime secretion of dehydroepiandrosterone (DHEA) in healthy female employees: cross-sectional analyses. *Sci. Rep.* **5**, 15844; doi: 10.1038/srep15844 (2015).

## Supplementary Material

Supplementary Figure S1 and Tables S2 and S3

## Figures and Tables

**Table 1:** General characteristics, job strain and social support scores and area under the curve with respect to ground (AUC_G_) of salivary dehydroepiandrosterone (DHEA), cortisol and cortisol-to-DHEA molar ratio (C/D ratio) (n = 115).

Variables	frequency (%)/mean (SD)
Age (years)	30.8 (8.5)
20–29	66 (57%)
30–39	26 (23%)
40–49	23 (20%)
Employment status	
Regular staff	85 (74%)
Contract worker	30 (26%)
Current smoking	2 (2%)
Menstruation irregularity	25 (22%)
Ovulatory phase	12 (10%)
Health disorders	
Musculoskeletal symptoms	75 (65%)
Dental and gum diseases	15 (13%)
Other health problems	8 (7%)
Job strain score	0.52 (0.08)
Social support score	25.6 (3.4)
AUC_G_, log-transformed1)	
DHEA	0.24 (0.61)
Cortisol	2.52 (0.59)
C/D ratio	4.08 (0.65)

SD: standard deviation.

^1)^AUC_G_ was log-transformed for normality.

**Table 2:** Age-adjusted mean (standard error) of salivary dehydroepiandrosterone (DHEA), cortisol and cortisol-to-DHEA molar ratio (C/D ratio) at 9:00, 12:00 and 15:00 (n = 115).

	9:00	12:00	15:00
DHEA (nmol/l)	0.23 (0.02)	0.28 (0.02)	0.21 (0.01)
Cortisol (nmol/l)	3.7 (0.3)	2.0 (0.1)	1.9 (0.1)
C/D ratio	19.9 (1.8)	8.9 (0.7)	11.3 (0.7)

**Table 3:** Matrix of Pearson correlation coefficients (n = 115).

	1	2	3	4	5	6	7
	Strain	Support	DHEA	Cortisol	C/D ratio	Age	Employment
Job strain score		−0.094	0.068	−0.097	−0.175	−0.057	0.010
Social support score			−0.196*	−0.158	0.034	−0.271**	−0.058
AUC_G_1) of salivary DHEA				0.396***	−0.596***	−0.469***	0.216*
AUC_G_1) of salivary cortisol					0.491***	−0.086	0.167
AUC_G_1) of salivary C/D ratio						0.388***	−0.055
Age							0.102
Employment status (regular staff = 1)							

DHEA: dehydroepiandrosterone. C/D ratio: cortisol-to-DHEA molar ratio. *P < 0.05, **P < 0.01, ***P < 0.001.

^1)^Area under the curve with respect to ground (AUC_G_) was log-transformed for normality.

**Table 4:** Job strain and social support scores and area under the curve with respect to ground (AUC_G_) of salivary dehydroepiandrosterone (DHEA), cortisol and cortisol-to-DHEA ratio (C/D ratio): results of multiple linear regression analysis (n = 115).

AUC_G_, log-transformed1)	Job strain score		Social support score		Goodness of fit		
	SPRC	P	SPRC	P	Adjusted R^2^	F	P
Model 1							
DHEA	0.000	0.999	−0.340	<0.0012)	0.375	18.074	<0.001
Cortisol	−0.128	0.166	−0.205	0.033	0.055	2.663	0.036
C/D ratio	−0.138	0.112	0.130	0.147	0.169	6.788	<0.001
Model 2							
DHEA	0.002	0.978	−0.343	<0.0012)	0.352	7.201	<0.001
Cortisol	−0.132	0.176	−0.181	0.084	0.008	1.094	0.374
C/D ratio	−0.145	0.111	0.155	0.111	0.136	2.787	0.004

SPRC: Standardized partial regression coefficient. In Model 1, the independent variables were job strain and social support scores, age and employment status (regular staff). The degree of freedom for F-value was 4, 110. In Model 2, the independent variables additionally included current smoking, menstruation irregularity, ovulatory phase, musculoskeletal symptoms, dental and gum diseases and other health problems. The degree of freedom for F-value was 10, 104.

^1)^AUC_G_ was log-transformed for normality.

^2)^Statistical significance remained (p < 0.001) even when log-transformed AUC_G_ of salivary cortisol was additionally adjusted (see Supplementary Table S3).
