# Supplementary material for: Psychological job strain, social support at work and daytime secretion of dehydroepiandrosterone (DHEA) in healthy female employees: cross-sectional analyses
Source: Sci Rep. 2015 Nov 10;5:15844. doi: 10.1038/srep15844 (PMC4639848; doi:10.1038/srep15844)
Supplement: Supplementary Figure S1 and Tables S2 and S3 [file srep15844-s1.pdf]

Psychological job strain, social support at work and daytime secretion of dehydroepiandrosterone (DHEA) in healthy female employees: cross-sectional analyses

Atsuhiko Ota[1*], Hiroshi Yatsuya[1], Junji Mase[1, 2] & Yuichiro Ono[1]

1. Department of Public Health, Fujita Health University School of Medicine, Toyoake, Japan
2. Division of Dentistry, Aichi Cancer Center Hospital, Nagoya, Japan

*Corresponding author's email address: ohtaa@fujita-hu.ac.jp (A.O.)

Supplementary information: Supplementary Figure S1 and Tables S2 and S3 are attached.

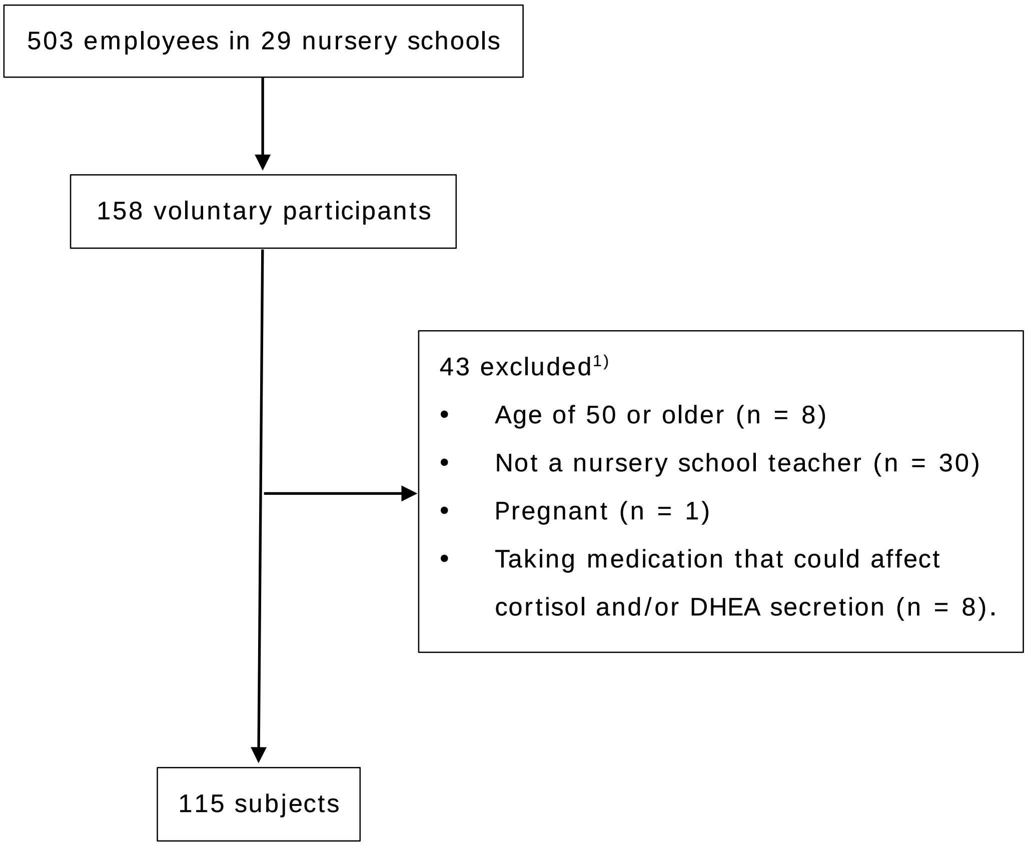

Supplementary Figure S1 | Flowchart of selecting the subjects

1) Some met more than one of the exclusion criteria.

Supplementary Table S2 | Matrix of Pearson correlation coefficients among job strain and social support scores, salivary dehydroepiandrosterone (DHEA), cortisol and cortisol-to-DHEA ratio (C/D ratio) and general characteristics (n = 115)

	1	2	3	4	5	6	7	8	9	10	11	12	13
1. Job strain		-0.094	0.068	-0.097	-0.175	-0.057	0.010	-0.058	0.131	0.003	-0.013	-0.211*	-0.094
2. Social support			-0.196*	-0.158	0.034	-0.271**	-0.058	-0.004	-0.083	-0.228*	-0.114	0.100	0.216*
3. DHEA[1)]				0.396***	-0.596***	-0.469***	0.216*	-0.016	0.127	-0.060	-0.092	0.026	-0.119
4. Cortisol[1)]					0.491***	-0.086	0.167	-0.040	0.044	0.057	-0.014	0.022	-0.094
5. C/D ratio[1)]						0.388***	-0.055	-0.020	-0.087	0.116	0.063	-0.014	0.032
6. Age							0.102	-0.013	-0.053	0.087	0.208*	-0.030	-0.011
7. Employment status[2)]								-0.224*	0.073	-0.121	0.107	-0.005	-0.071
8. Current smoking[3)]									0.091	-0.045	0.097	0.146	0.225*
9. Menstruation irregularity[3)]										-0.180	0.119	-0.142	0.022
10. Ovulatory phase[3)]											-0.049	-0.048	-0.093
11. Musculoskeletal symptoms[3)]												0.120	-0.016
12. Dental and gum diseases[3)]													-0.004
13. Other health problems[3)]													

* P < 0.05, ** P < 0.01, *** P < 0.001.

1) Presented as log-transformed area under the curve with respect to ground (AUC_G). Log-transformation was done for normality.

2) Regular staff = 1, Contract worker = 0

3) Applicable = 1, Not applicable = 0

Supplementary Table S3 | Social support scores and area under the curve with respect to ground (AUC_G) of salivary dehydroepiandrosterone (DHEA): results of multiple linear regression analyses (n = 115)

AUC_G, log-transformed[1)]	Social support score		Goodness of fit		
	SPRC	P	Adjusted R^2	F	P
Model 1					
DHEA	-0.284	< 0.001	0.440	18.906	< 0.001
Model 2					
DHEA	-0.294	< 0.001	0.420	8.507	< 0.001

SPRC: Standardized partial regression coefficient. In Model 1, the independent variables were job strain and social support scores, age, employment status (regular staff) and log-transformed AUC_G of cortisol. The degree of freedom for F-value was 5, 109. In Model 2, the independent variables additionally included current smoking, menstruation irregularity, ovulatory phase, musculoskeletal symptoms, dental and gum diseases, other health problems and log-transformed AUC_G of cortisol. The degree of freedom for F-value was 11, 103.

1) AUC_G was log-transformed for normality.
